# 

**Published:** 1875-04

**Authors:** 


### CONTENTS.

PAGE.

**CONTRIBUTIONS.**

Brains vs Bowels. Ben. H. Pratt........ 1

Waste of Life. P. H. Hayes, M. D........ 3

Salvation—Health. Rev. Thos. K. Beecher........ 4

**MEDICAL ITEMS AND NEWS.**

Bedsores — Enlargement of Spleen — Epistaxis — Coryza—Chilblains and Frosted Feet — Dropsy of Joints—Iodide of Potassium in Diphtheria—Asthma — Ozæna — Painful Hemorrhoids—Secondary Syphilis — Hydrocele — Diphtheria — Pityriasis Capitis — Chronic Hepatitis—Dyspepsia Pills—Recurrent Chronic Bronchitis — Catarrh and Paralsis of Bladder — Itch — Diagnostic Hint in old Ulcers—Cough Mixture— Biliary Calculi — Rheumatism —Cinquefoil in Peritonitis— Syrup Hypophosphate Iron, Quinia and Strychnia — Gonorrhœa — Veratrum in Laringitis — Iced Clysters in Dysentery—Neuralgia of Larynx—Burns—Dyphtheria—Seasickness— Hysterical Spasms -- Pneumonia and Bronchitis — Polk's Syrup Ferrous Hypophosphite Comp.— Dilate or Cut—Preservation and use of Vaccine Virus — Ulceration of Nose in Scrofulous Children — Bromide of Ammonium in Rheumatism— Hay Fever — Sycosis — Inverted Toe Nails — Injection of Chloral in Trachia — Neuralgia — Acute and Chronic Bronchitis and Asthma — Catarrh — Washing out the Stomach — Bromide of Iron in Chorea........6 to 11

**OUR CORRESPONDENCE.**

Fourteen Letters and Replies........12 to 13

**CLIPPINGS.**

Sleeplessness—A Darwinian Plea........ 5
Advice to a Dyspeptic........ 5
Diphtheria........18
To keep Ice—Dogs and Books as Vehicles of Disease —Scarlet Fever by Mail -- Dipsomania— Quick Tanning........19

PAGE.

**HOUSEHOLD HINTS AND RECIPES.**

Chilblains—Water Proof Cloth— Restoring Ivory — Poisonous Chewing Gum—Chilblains — Imitation of Tortoise Shell— To Clean Woodwork — Glue to Resist Fire — Viennese Meerschaum— Repairs on Plastered Walls — Transparent Pomade — Liquid Slating — German Apple Pudding —Potatoes a la Duchesse—Moth Preventive —To Cook Apples — Bruises in Furniture — To give Iron Wire Silvery Look—Chapped Hands— Simple Plan of Ventilation—To Coat Iron with Brass — Cleaning Brass— Red Marking Ink—Stains for Wood — To Cement Metal on Glass — Writers' Cramp— Remedies for Stings—Strychnia Antidote—Cement for Labels— Soldering Broken Files—Novel Rat Trap—Apples in Imitation of Ginger—Bottling Cider........14 to 17

**EDITORIAL.**

Criminal Negligence........20
Ectropium........20
Deformities of the Feet........21
Deformities of the Toes........22
Look Out........23
Spring Fever........24
Take your Vacation........24

**EDITORIAL CHIT-CHAT.**

Ex Mayor Caldwell—Pills—Behind Time—Religious Insanity—July Number—Livingstone's Humerus— Cork in California—Dissecting Room Scene—A Difficult Order—Drunken Babies—Photograph their Hands—A Reason for It—Co-Education of the Sexes — Infinitesimal Dosing — Manifestations— Chloroform Death—A Difference in Medical Views —Curiosity—Disagreeable—That Winter—Grateful—Patent Tent—School Hygiene—Our Improvements—The Fair—"There's Millions in It"—Medical Advertising—New Books and Periodicals 25 to 28

